# *ATG9A* Is Overexpressed in Triple Negative Breast Cancer and Its In Vitro Extinction Leads to the Inhibition of Pro-Cancer Phenotypes

**DOI:** 10.3390/cells7120248

**Published:** 2018-12-06

**Authors:** Aurore Claude-Taupin, Leïla Fonderflick, Thierry Gauthier, Laura Mansi, Jean-René Pallandre, Christophe Borg, Valérie Perez, Franck Monnien, Marie-Paule Algros, Marc Vigneron, Pascale Adami, Régis Delage-Mourroux, Paul Peixoto, Michael Herfs, Michaël Boyer-Guittaut, Eric Hervouet

**Affiliations:** 1 INSERM, EFS BFC, UMR1098, Interactions Hôte-Greffon-Tumeur/Ingénierie Cellulaire et Génique, University Bourgogne Franche-Comté F-25000 Besançon, France; taupin.aurore@gmail.com (A.C.-T.); leila.fonderflick@orange.fr (L.F.); thierry.gauthier59@hotmail.fr (T.G.); mansi.laura@gmail.com (L.M.); jean-rene.pallandre@efs.sante.fr (J.-R.P.); christophe.borg@efs.sante.fr (C.B.); valerie.perez@univ-fcomte.fr (V.P.); pascale.adami@univ-fcomte.fr (P.A.); regis.delage-mourroux@univ-fcomte.fr (R.D.-M.); paul.peixoto@univ-fcomte.fr (P.P.); michael.boyer-guittaut@univ-fcomte.fr (M.B.-G.); 2 Department of Pathology, University Hospital of Besançon, F-25000 Besançon, France; fmonnien@chu-besancon.fr (F.M.); mp1algros@chu-besancon.fr (M.-P.A.); 3 Team Replisome Dynamics and Cancer. UMR7242 Biotechnologie et Signalisation Cellulaire, CNRS-University Strasbourg, F-67412 Illkirch, France; marc.vigneron@unistra.fr; 4 Ecole Supérieure de Biotechnologie de Strasbourg, University Strasbourg, CNRS, UMR 7242, F-67412 Illkirch, France; 5 EPIGENEXP platform, University of Bourgogne Franche-Comté, F-25000 Besançon, France; 6 Boratory of Experimental Pathology, GIGA-Cancer, University of Liege, B-4000 Liege, Belgium; m.herfs@ulg.ac.be; 7 DimaCell platform, Univ. Bourgogne Franche-Comté, F-25000 Besançon, France

**Keywords:** ATG9A, autophagy, triple negative breast cancer, MDA-MB-436, shRNA, CRISPR/Cas9

## Abstract

Early detection and targeted treatments have led to a significant decrease in mortality linked to breast cancer (BC), however, important issues need to be addressed in the future. One of them will be to find new triple negative breast cancer (TNBC) therapeutic strategies, since none are currently efficiently targeting this subtype of BC. Since numerous studies have reported the possibility of targeting the autophagy pathway to treat or limit cancer progression, we analyzed the expression of six autophagy genes (*ATG9A*, *ATG9B*, *BECLIN1*, *LC3B*, *NIX* and *P62/SQSTM1*) in breast cancer tissue, and compared their expression with healthy adjacent tissue. In our study, we observed an increase in *ATG9A* mRNA expression in TNBC samples from our breast cancer cohort. We also showed that this increase of the transcript was confirmed at the protein level on paraffin-embedded tissues. To corroborate these in vivo data, we designed shRNA- and CRISPR/Cas9-driven inhibition of *ATG9A* expression in the triple negative breast cancer cell line MDA-MB-436, in order to determine its role in the regulation of cancer phenotypes. We found that *ATG9A* inhibition led to an inhibition of in vitro cancer features, suggesting that ATG9A can be considered as a new marker of TNBC and might be considered in the future as a target to develop new specific TNBC therapies.

## 1. Introduction

Breast cancer (BC) is one of the three most commonly diagnosed cancers in women. In 2018, 266,120 new cases of female invasive BC were diagnosed in the United States [1]. Thanks to earlier detection and the development of specific targeted treatments, mortality has declined by about 30% over the past two decades [2]. Nevertheless, the American Cancer Society still estimated 41,400 deaths linked to breast cancer in the United States in 2018 [1], rendering it a major health problem. Triple negative breast cancer (TNBC) represent approximately 10% of BCs and is defined as tumors lacking estrogen receptor (ER), progesterone receptor (PR), and Human Epidermal Growth Factor Receptor 2 (HER2) expression (ER^−^, PR^−^, HER2^−^). TNBC presents with a poor prognosis mainly because of the limited treatment options. In fact, no specific therapy targeting TNBC is currently available, and cytotoxic chemotherapy remains the current treatment strategy [3]. 

During the course of development of new therapeutic options for the treatment of BC, numerous studies have reported the possibility of targeting the autophagy pathway. For example, autophagy inhibition has been shown to improve chemotherapy efficiency in TNBC presenting with high levels of LC3B expression, the main protein marker of autophagy [4], and to enhance the therapeutic response in both anthracycline-sensitive and resistant TNBC cells [5]. Autophagy has also been proposed to protect TNBC cells from damaged mitochondria accumulation induced by chemotherapies [6]. These data led to the publication of several phase II clinical trials, which were conducted, or are underway, to determine the efficiency and safety of hydroxychloroquine, an autophagy inhibitor preventing lysosomal acidification, in combination with different chemotherapies (Ixabepilone, Plaquenil, Everolimus, and Gedatolisib) in recurrent or metastatic breast cancer patients (NCT00765765, NCT02414776, NCT03032406, NCT03400254, *clinicaltrials.gov (US National Library of Medicine*).

Autophagy is a cellular process involved in the maintenance of cell homeostasis and cell survival, by inducing the degradation and/or the recycling of intracellular components such as protein aggregates or organelles (e.g., damaged mitochondria). This process is mediated by more than 40 core ATG (AuTophaGy-related) proteins and characterized by the formation of a double-membrane structure, called phagophore, which elongates and closes to generate an autophagosome. The autophagosome, which engulfs cytosolic material as well as organelles, later fuses with a lysosome to form an autophagolysosome, leading to the degradation of its content by lysosomal proteases (reviewed in [7]). The initiation and elongation of this structure requires several ATG proteins, one of them being the ATG9 protein. Two isoforms of ATG9, A and B, are expressed in mammals and these proteins are the only transmembrane ATG proteins identified so far. ATG9A is ubiquitously expressed, while ATG9B is enriched in the placenta and pituitary gland, suggesting that ATG9B may play a specific role during embryogenesis [8]. While the specific function of these two isoforms remains unclear, it has been proposed that ATG9B may complement ATG9A during autophagy. ATG9A presents six conserved transmembrane domains with both N- and C-terminus localized on the cytofacial side of the vesicle. ATG9A continuous cycling between the plasma membrane, endosomes and trans-Golgi network is believed to be necessary for a Futophagosome formation and vesicles containing ATG9A have therefore been proposed as the main candidates for delivering lipids to growing phagophores [9] Indeed, under nutrient-rich conditions, ATG9A is found in the trans-Golgi network, recycling and late endosomal compartments [9,10,11,12,13,14,15], but upon amino-acid starvation, it is relocalized towards the autophagosome initiation site, where it dynamically interacts with phagophores and autophagosomes, without being incorporated into them [10]. 

ATG9A dysregulation has already been linked to cancer, since ATG9A protein overexpression has been associated with poor survival in patients with oral squamous cell carcinoma [16], whereas ATG9A inhibition was associated to the apparition of Trastuzumab resistance in HER2^+^ BC cells [17]. Furthermore, a recent study reported a decrease in *ATG9A* mRNA expression in breast invasive ductal carcinomas, compared to adjacent healthy tissues, and this decrease was linked with increased promoter methylation [18]. More interestingly, the authors also demonstrated a significant decrease in *ATG9A* mRNA expression in HER2^+^ BC, but to our knowledge, no published study has already characterized the expression and the role of ATG9A in TNBC.

In this study, we therefore analyzed the expression of six *ATG* genes (*ATG9A*, *ATG9B*, *BECLIN1*, *LC3B*, *NIX* and *SQSTM1/P62*) using quantitative real-time PCR (qRT-PCR) in BC tissues, and compared their expression with healthy adjacent tissue. Our data clearly demonstrated that only *ATG9A* mRNA levels were significantly higher in TNBC tissue compared to healthy adjacent tissue. We also confirmed these data at the protein level using Immunohistochemical (IHC) analysis of tumors compared to healthy adjacent tissues. We then inhibited *ATG9A* expression in the TNBC cell line MDA-MB-436, and observed that *ATG9A* inhibition led to an inhibition of in vitro cancer features, such as proliferation and invasion.

## 2. Materials and Methods

### 2.1. Ethics Statement

Human samples were collected according to French laws, and the recommendations of the French National Committee of Ethics. Indeed, this study has been approved by the scientific committee of the “Tumorothèque Régionale de Franche-Comté” (BB-0033-00024) in 2003. The samples and the medical history of patients were encoded to protect patient confidentiality, and used under protocols approved by the recommendations of the French national Committee of Ethics. All human samples were collected by Pr. Séverine Valmary-Degano (Centre Hospitalier Régional Universitaire, Besançon, France) at the “Tumorothèque régionale de Franche-Comté”. Collection of samples and their use (AC-2010-1163) for further studies have been approved by the French “Ministère de la Recherche” and by the CPP EST II. We obtained all required consents from any patients involved in the study.

### 2.2. Tumor Samples

Patients were treated in two medical centers (“Centre Hospitalier Régional Universitaire de Besançon” and “Centre Hospitalier Régional de Belfort-Montbéliard”), and the samples collected were included by the “Tumorothèque Régionale de Franche-Comté” (BB-0033-00024) between January 2007 and September 2014 (*n* = 80). The median age was 58 years (range 26–85) and patients were selected according to their BC subtype at the time of diagnosis. Breast tumor subtypes were determined using ER (estrogen receptor), PR (progesterone receptor), and HER2 (human epidermial growth factor receptor 2) immunohistochemistry (IHC) staining (paraffin-embedded material). The threshold of negative signs was classified for ER and PR at ≤10% regardless of the staining intensity. Staining of HER2 was divided into negative (0/1+) and positive (2/3+), where 2+ staining was confirmed using SISH (silver in situ hybridization) amplification. The histological grade was determined according to the Elston and Ellis classification [19]. All the clinical information regarding the different patients included in the study can be found in Table 1.

### 2.3. Antibodies

For western blotting experiments or IHC, the following antibodies were used: Rabbit monoclonal anti-ATG9A (Abcam, Paris, France, ab108338), rabbit anti-KI67 (Ventana, clone 30-9,), rabbit anti-LC3B (Sigma-Aldrich, Saint-Quentin Fallavier, France, L8918), rabbit polyclonal anti-ACTIN (Sigma-Aldrich, A5060), and secondary goat polyclonal anti-rabbit HRP (Abliance, Compiègne, France, BI2407).

### 2.4. Cell Culture

MDA-MB-231 and MDA-MB-436 cells were cultured in Dulbecco’s minimum essential medium (DMEM) (Dutscher, 67170 Brumath France, L0066) supplemented with 100 μg/mL penicillin/streptomycin (Dutscher, L0018), 10% fetal bovine serum (FBS; Dutscher, S1810), and 0.4 mg/L amphotericin B in a 5% CO_2_ atmosphere at 37 °C. Bafilomycin A1 (Sigma-Aldrich, B1793) was used at 500 nM for 2 h, and 3-methyladenine (Sigma-Aldrich, M9281) was used at 2.5 mM for 3 days.

### 2.5. Stable Cell Lines

To create the MDA-MB-436-shRNA-Control (1 and 2), –*ATG9A* (A, B, and C), and MDA-MB-231-shRNA-*Control* (I and II)–*ATG9A* (a and b) cell lines, MDA-MB-436 or MDA-MB-231 cells (340,000) were plated onto 6-well plates. The day after plating, cells were transfected using 2 µg of pLKO.1-puro-shRNA-Control vector (Mission non-mammalian shRNA control plasmid, Sigma Aldrich, SHC002), or pLKO.1-puro-shRNA-*ATG9A* vector (Mission shRNA, Sigma Aldrich, SHCLNG-NM_024085.3-3318s21c1) and 4 µL of Jetprime reagent (Polyplus, 114-07), according to the manufacturer’s protocol. Two days later, puromycin (5 µg/mL) was added to the cells, and the medium was changed every 2 days for the next 14 days until the detection of puromycin-resistant single clones. Each clone was then tested for the expression of *ATG9A* mRNA by RT-qPCR, and protein by western Blotting. The stable cell lines, which were then used in this study, were the MDA-MB-436-shRNA-*Control* clones (1 and 2), the MDA-MB-436-shRNA-*ATG9A* clones (A, B and C), the MDA-MB-231-shRNA-*Control* clones (I and II), and the MDA-MB-231-shRNA-*ATG9A* clones (a and b).

To create the MDA-MB-436-CRISPR-Control and -*ATG9A* cell lines, MDA-MB-436 cells (340,000) were plated onto 6-well plates. The day after plating, cells were transfected using 2 µg of pSpCas9(BB)-2A-GFP-*Control* vector (Addgene, 48138) or pSpCas9(BB)-2A-GFP-*ATG9A* (sgRNA sequence: TGCCCTTCCGTATTGCACG), and 4 µL of Jetprime reagent (Polyplus, 114-07), according to the manufacturer’s protocol. One day later, GFP-positive cells were sorted using a Sony cell sorter (SH800S) and plated onto 6-well plates (500 cells/well) until the detection and the characterization of single clones, as described above for the shRNA clones. The stable cell lines which were then used in this study were the MDA-MB-436-CRISPR-*Control* and the MDA-MB-436-CRISPR-*ATG9A*.

### 2.6. Western Blotting

Cells were scraped and harvested in cold phosphate-buffered saline (PBS; 137 mM NaCl, 2.7 mM KCl, 10 mM Na_2_HPO_4_, and 1.8 mM KH_2_PO_4_), and lysed in SB1X (45 mM Tris-HCl, pH 7.6, 10% Glycerol, 2% SDS (Sodium Dodecyl Sulfate), 1.5% 2-mercaptoethanol, and 0.001% bromophenol blue). Protein lysates were sonicated for 10 s (Sonics and Materials, Vibra-Cell) before loading (20 µg), and then separated on a 10 or 12.5% polyacrylamide gel in running buffer (25 mM Tris base, 192 mM Glycine, and 1% SDS) at 20 mA for 80 min. The gel was then transferred onto a polyvinylidene difluoride (PVDF) 0.2 µm membrane (Bio-Rad, 162-0177) at 200 mA for 2 h in transfer buffer (25 mM Tris base, 192 mM Glycine, and 10% methanol). The membrane was blocked with 5% non-fat milk in Tris-buffered saline supplemented with Tween 20 (TBS-T; 20 mM Tris base, pH 7.6, 137 mM NaCl, and 0.1% Tween 20) for 1 h and incubated with primary antibodies in TBS-T supplemented with 1% non-fat milk, overnight at 4 °C. Following three washes in TBS-T, immunoreactive bands were detected using a secondary goat horseradish peroxidase (HRP)-coupled secondary anti-rabbit antibody and the Clarity Western ECL blotting substrate (Biorad, Marnes-la-Coquette, France, 1705061). The signals were then analyzed and quantified using the ChemiDoc XRS+ system (Biorad), and the Image Lab software (5.1, Biorad).

### 2.7. RNA Extraction and RT-qPCR

Total RNA were extracted using the Tri Reagent (Molecular Research Center, TR-118) as previously described [20]. Briefly, the cell pellet (300,000 cells) was suspended in 750 µL of Tri Reagent, and 0.2 mL of chloroform was added before centrifugation at 12,000× *g* for 15 min at 4 °C. Total RNA was then precipitated using 0.5 mL of isopropanol and centrifuged at 10,000× *g* for 20 min at 4 °C. The RNA pellet was washed with 70% ethanol before solubilization in water (30 µL) and then incubated for 10 min at 65 °C. Total purified RNA was then quantified before conservation at −80 °C. 

For RNA extraction from patient samples, 20 mg of breast tissue (tumor or healthy adjacent tissue) was reduced in liquid nitrogen before homogenization in 750 µL of Tri Reagent for 15 min at 30 °C, and centrifugation at 2550× *g* for 2 min in order to eliminate fat tissue. 

For RT-qPCR analysis, 1.5 µg of total RNA was reverse transcribed using the M-MLV Reverse Transcriptase (Sigma, M1302), 1.25 µM of oligo(dT) 23 (Eurogentec), and 1.25 µM of random primers (Promega, Charbonnières-les-Bains, France C1181). Quantitative PCR was run on the Step One Real Time PCR System (Fisher Scientific, Illkirch, France) using the Syber Green PCR Master Mix (Applied Biosystems, 4309155) and the following parameters: 10 min at 95 °C followed by 40 cycles; 15 s at 95 °C and 1 min at 60 °C. The target gene levels (*ATG9A*, *ATG9B*, *BECLIN1*, *LC3B*, *NIX*, and *SQSTM1*) for tissues were normalized to the ratio of expression from two housekeeping genes: *RPLP0* and *18S rRNA*. The target gene levels for cell samples (*ATG9A*) were normalized to the *18S rRNA* gene expression. The primer sequences used for qPCR analysis are described in the Table 2. Each sample was analyzed in duplicate and then differences in the expression of each gene were quantified using the standard curve method using plasmids containing the cloned target sequences as controls for absolute quantification.

### 2.8. Cell Proliferation Assays

For the MTT assay (3-(4,5-dimethylthiazol-2-yl)-2,5-diphenyl tetrazolium bromide; Sigma-Aldrich, M5655), cells were plated in 96-well plates (3,000 cells per well, 16 wells per cell line) and MTT assays were conducted every day over a 4 day period as previously described [20]. Briefly, 100 µL of a 0.5 mg/mL MTT solution, diluted in sterile PBS, was added in each well, then incubated for 2 h in 5% CO_2_ at 37 °C. Following a 5 min centrifugation at 500× *g*, the MTT solution was discarded, and 50 µL of Dimethyl sulfoxide (DMSO) was added to each well. Then, the absorbance was measured at 549 nm with a reference wavelength at 620 nm using a microplate reader (Thermo Scientific, Illkirch, France, Multiskan FC).

For the trypan blue exclusion assay, cells were plated in 24-well plates (30,000 cells per well, 2 wells per cell line) and counted every day over a 4 day period. After discarding the culture medium, cells were incubated in 100 µL Trypsin-EDTA (Dutscher, X-0930) for 5 min at 37 °C before the addition of 200 µL of complete medium and centrifugation at 500× *g* for 5 min. The cell pellet was then suspended in 50 µL of trypan blue (0.04% in PBS, Corning, 702630) and the number of viable cells was then determined using a Malassez haemocytometer.

### 2.9. Cell Migration and Invasion Assays

Cell migration was quantified using a culture-insert 2 well in 35 mm microdishes (81176, Ibidi). In each well, 18,000 cells were plated and the insert was removed 24 h later. The migration rate was then evaluated 24 h later.

Cell invasion was evaluated using modified Boyden chambers (ThinCert for 24 well plates, 8 µm pore size, Greiner Bio-one, 438122). Boyden chambers were coated with 50 µg ECM (extra cellular matrix) gel from Engelbreth-Holm-Swarm murine sarcoma (Sigma-Aldrich, E1270) diluted in DMEM for 5 h at 37 °C. A total of 100,000 cells in 250 µL of serum-free DMEM were seeded into the upper chamber and 500 µl of complete culture medium was added in the lower compartment. After a 24 h incubation in a 5% CO_2_ atmosphere at 37 °C, cells in the upper compartment were removed using a swab, and cells in the lower compartment were fixed for 5 min with absolute ethanol, then stained with crystal violet (0.5% in 2% ethanol) for 10 min. The filters were then washed with distilled water and cell density was quantified using the threshold color plugin of the ImageJ software. 

### 2.10. Immunohistochemistry and Immunostaining Assessment

Immunohistochemical (IHC) analysis was performed using a standard protocol previously detailed [21]. Staining intensities were then quantified using the intensity quantification method (score: 0–3 and extent: 0–3). As previously described [22], the 2 scores were then multiplied to obtain an overall score ranging between 0 and 9. All immune-labeled tissues were evaluated by two experienced histopathologists.

### 2.11. Statistical Analysis

Statistical analyses were performed using the student t test. Data were expressed as the mean ± SEM. * *P* ≤ 0.05; ** *P* ≤ 0.01 and *** *P* ≤ 0.001.

## 3. Results

### 3.1. ATG9A Expression Was Increased in Breast Cancers

**Figure 1:**
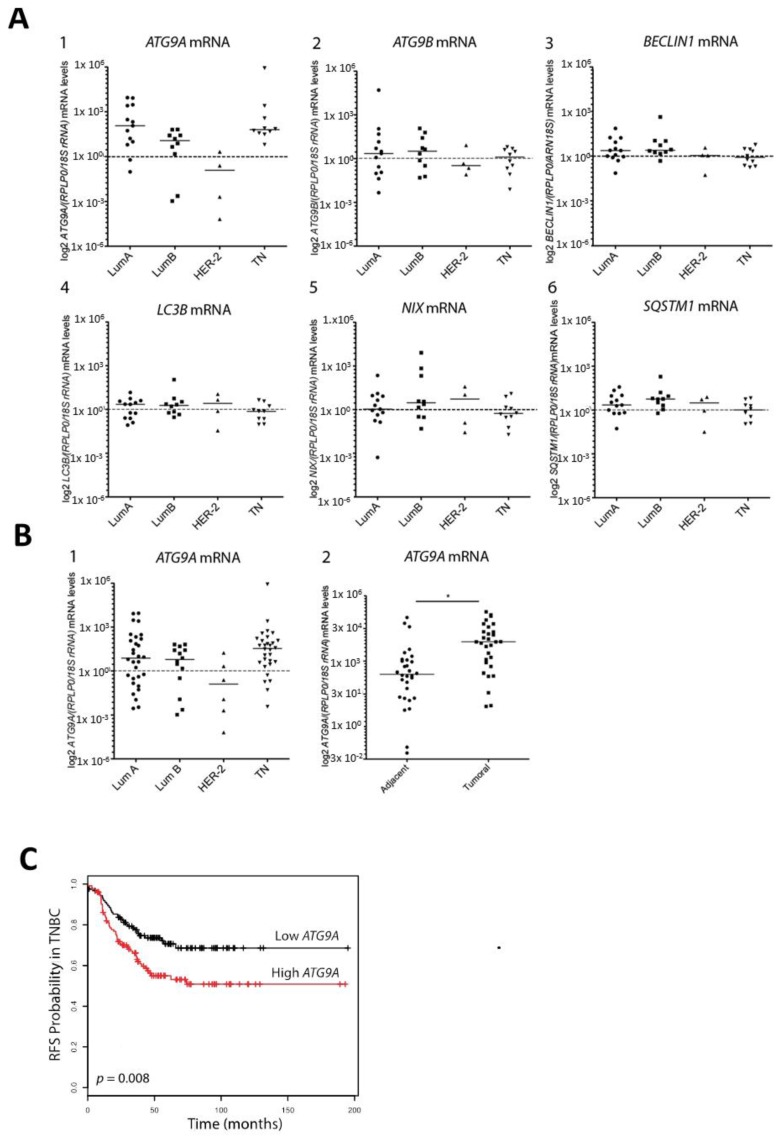
ATG9A mRNA overexpression in triple negative breast cancer. (**A**) Quantification of *ATG9A*, *ATG9B*, *BECLIN1*, *LC3B*, *NIX*, and *SQSTM1* mRNA levels using RT-qPCR, in Luminal A (LumA), Luminal B (LumB), HER2, and triple negative (TN) breast cancer biopsies compared to matched healthy adjacent tissues (*n* = 37). Values were normalized to the ratio of two housekeeping genes, *rRNA18S* and *RPLP0*. Data presented on scatter plots represent the log2 of *ATG9A*, *ATG9B*, *BECLIN1*, *LC3B*, *NIX*, and *SQSTM1* mRNA levels ratio in breast cancer biopsies over healthy adjacent tissues, for each patient. (**B**) The Left panel represents the ratio of *ATG9A* mRNA levels in LumA, LumB, HER2, and TN biopsies compared to matched healthy adjacent tissues (expanded cohort, *n* = 80). Data are expressed in log2 of relative *ATG9A* mRNA expression, for each patient, normalized with the ratio of two housekeeping genes: *rRNA18S* and *RPLP0*. The Right panel represents the log2 of *ATG9A* mRNA levels in healthy adjacent tissues (left) and TN biopsies (right), normalized to the ratio of two housekeeping genes, *rRNA18S* and *RPLP0*. Difference of expression was quantified using a paired t-test (* *p* = 0.0325). (**C**) Kaplan–Meier curves representing relapse-free survival (RFS) in TN patients expressing high (*n* = 125) or low (*n* = 124) *ATG9A* mRNA levels (*p* = 0.008; source: Kmplot.com).

The different BC subtypes are classified depending on their molecular marker expression. Luminal A and Luminal B, which represent 70% of total BCs [19], are generally associated with a good prognosis and are characterized by ERα expression (ER^+^), whereas the amplification of the *HER2* gene is only observed in the Luminal B subtype. The HER2^+^ BC subtype, which represents 10–15% of overall BCs, presents an amplification of the *HER2* gene without the expression of ERα (ER^−^). TNBCs, which present no expression of ER, PR, or HER2, generally exhibit poor outcomes. Consequently, TNBCs present a significant challenge, and molecular-profiling efforts are under progress in order to determine new promising targets. Since autophagy has been reported to be involved in TNBC chemotherapy efficiency, we decided to analyze the expression of several *ATG* genes, in order to determine whether *ATG* gene expression was deregulated in BC. To this end, we first analyzed *ATG9A*, *ATG9B*, *BECLIN1*, *LC3B*, *NIX*, and *SQSTM1* mRNA levels in 37 human BC biopsies and compared their expression with the ones detected in healthy adjacent tissues, using qRT-PCR (Figure 1A). The clinical information regarding the patients whose samples have been used in our study can be found in Table 1. Our initial cohort included 13 Luminal A, 10 Luminal B, 4 HER2^+^, and 10 TN (triple negative) biopsies. Our results revealed no major difference in *ATG9B*, *BECLIN1*, *LC3B*, *NIX*, and *SQSTM1* mRNA expression between tumor and healthy adjacent tissues, but we observed an increase in *ATG9A* mRNA expression in Luminal A, Luminal B and TN patients, even if the significance of these differences remained to be confirmed. In order to validate this first set of data, we analyzed *ATG9A* mRNA levels in a larger cohort of 80 patients including 30 Luminal A, 14 Luminal B, 6 HER2^+^, and 30 TN (Figure 1B). The results obtained confirmed a significant increase in *ATG9A* mRNA levels in tumors compared to healthy adjacent tissues (*p* = 0.0355). To date, only seven out of the 80 patients from our cohort presented a cancer recurrence, and amongst them, five presented an increase in *ATG9A* mRNA levels in tumor cells compared to the healthy adjacent tissues (3/5 patients were diagnosed with TN, and 2/5 with Luminal B). However, due to the low number of patients presenting a cancer recurrence, we were not able to correlate the risk of recurrence to the difference of *ATG9A* expression levels between patients presenting a low or a high *ATG9A* mRNA expression. To establish the relevance of a link between *ATG9A* expression levels and clinical outcome in TNBC, we used the online survival analysis tool “KM plotter” [23], which integrates numerous gene expression data and survival information derived from 3,951 patients from databases such as The Cancer Genome Atlas (TCGA). The correlation of *ATG9A* expression levels with relapse-free survival (RFS) in TNBC was determined (Figure 1C), and we observed that high *ATG9A* levels correlated with shorter RFS, suggesting a link between *ATG9A* mRNA expression levels and survival of patients presenting a TNBC subtype.

**Figure 2:**
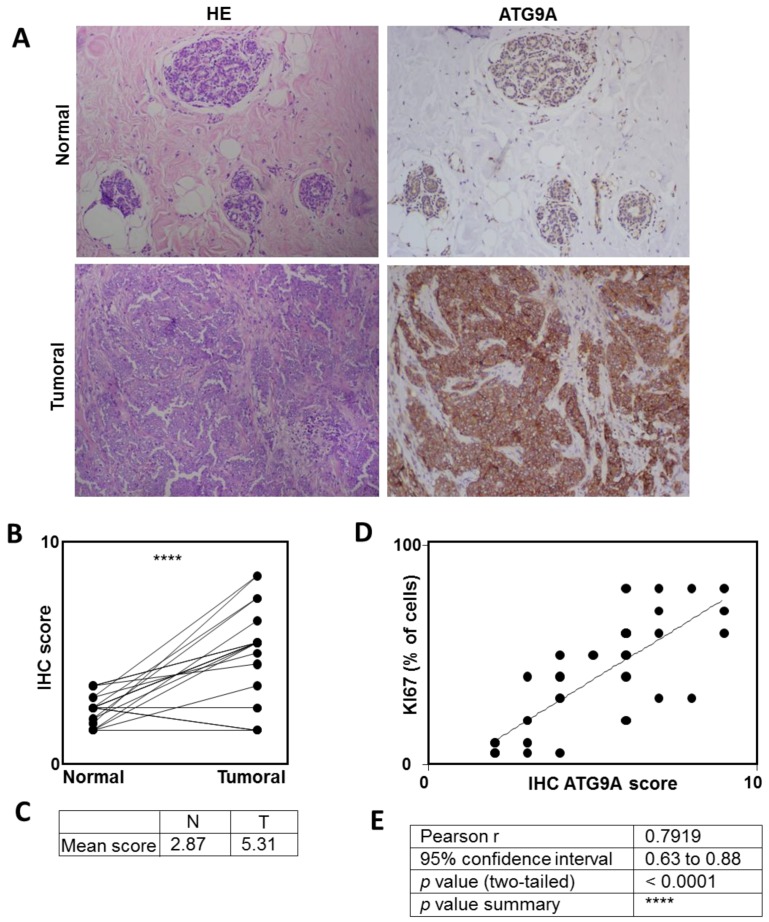
ATG9A protein overexpression in triple negative breast cancer. Quantification of ATG9A protein levels using IHC in triple negative breast cancer (TNBC) biopsies compared to matched healthy adjacent tissues. (**A**) Representative images of ATG9A protein staining in tumor and healthy tissues (left: HE (hematoxylin & eosin) staining, right: ATG9A staining). (**B**,**C**) Quantification of ATG9A staining in 21 patients. (**D**) Correlation between ATG9A score with KI67 staining in tumors. (**E**) Quantification.

We next decided to analyze whether the expression of the ATG9A protein was also up-regulated in TNBC. To do so, we analyzed the expression of the ATG9A protein in eight TNBC biopsies and compared its expression in the tumor compared to the healthy adjacent tissue. As expected, the ATG9A staining was significantly higher in 15 out of 21 patients (71%; *p* < 0.0001), four were unchanged, and only two were decreased in the tumor cells compared to the normal adjacent tissue (mean expression of 2.87 and 5.3 in normal and tumoral tissues, respectively; Figure 2). Interestingly, analysis of ATG9A protein expression in five TNBC cell lines showed that this protein was indeed expressed in all cell lines tested (Figure S1). When we compared the ATG9A score with the percentage of tumor cells expressing the proliferative marker KI67, we observed a strong correlation between ATG9 and KI67 (*p* < 0.001, and Pearson r 0.79; Figure 2C,D). Although the increase in *ATG9A* mRNA levels in LumA tumors compared to healthy adjacent tissues was less important than those observed with the TNBC patients, we also compared the expression of ATG9A protein in LumA and normal adjacent tissues (Figure S2A). We confirmed that ATG9A expression also significantly (*p* < 0.05) increased in LumA, but this was only observed in 10 out of 18 patients (55%). However, this increase was less important (*p* < 0.05) than whatwe observed in TNBC (*p* < 0.001), suggesting that the highest expression of *ATG9A* is in TNBC.

### 3.2. Inhibition of ATG9A Expression Using shRNA Inhibits In Vitro Cell Proliferation and Invasion

**Figure 3:**
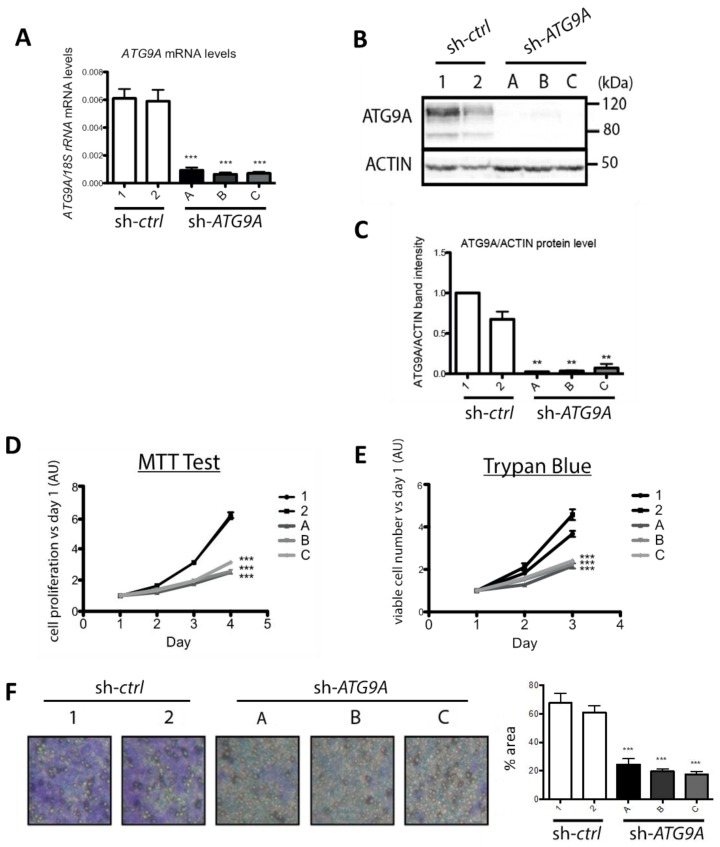
Inhibition of ATG9A expression using shRNA inhibits in vitro cell proliferation and invasion in the TNBC cell line MDA-MB-436. (**A**) ATG9A mRNA expression levels quantified by RT-qPCR in stable MDA-MB-436 clones expressing a sh-*control* (1 and 2) or a sh-*ATG9A* (A, B, and C; *n* = 3, duplicate). Values were normalized using the housekeeping gene *rRNA18S*. Difference in expression was quantified using a t-test: Compared to sh-*control* 1, *p* < 0.0001 for all sh-*ATG9A* clones (A, B, and C); compared to sh-*control* 2, *p* = 0.0002 for sh-*ATG9A* clone A and *p* < 0.0001 for sh-*ATG9A* clones B and C. (**B**) Levels of ATG9A and ACTIN proteins in the sh-*control* (clones 1 and 2) or sh-*ATG9A* (clones A, B, and C) clones. A representative image of three independent experiments is shown. (**C**) Quantification of ATG9A protein levels normalized to ACTIN protein levels, quantified using the ImageLab software (*n* = 3). The difference in expression was determined using a t-test: compared to sh-*control* 2, *p* = 0.0023; *p* = 0.0024, and *p* = 0.0048 for sh-*ATG9A* clones A, B, and C, respectively. ** *p* ≤ 0.01. Proliferation rates of sh-*ATG9A* and their controls using a MTT (**D**), or a Trypan-blue exclusion assay (**E**). The MTT assay was conducted every day over a four-day period (*n* = 3, 16 replicates). The difference in cell proliferation was quantified using a t-test at day 4. *** *p* ≤ 0.001 * *p* ≤ 0.05. For the trypan blue exclusion assay, viable cells were counted every day over a three-day period (*n* = 3, four replicates). The difference in cell number versus day 1 was quantified using a t-test at day 3 (graphs on the right). (**F**) The invasion assay was performed using sh-*ATG9A* (clones A, B, and C) and control clones (1 and 2). A representative image of three independent experiments with duplicates is shown on the left. The graph on the right represents the percentage of cells that migrated through the ECM-coated Boyden chambers after 24 h, quantification was done with the ImageJ software. The difference in cell density was quantified using a t-test. *** *p* ≤ 0.001.

In order to determine whether the high *ATG9A* mRNA levels quantified in TN tumors were a cause or a consequence of tumor development, we decided to inhibit *ATG9A* expression in the TNBC cell line MDA-MB-436. First, we transfected MDA-MB-436 cells with the pLKO.1-puro-shRNA-*ATG9A* vector (Mission shRNA, Sigma Aldrich), which encodes a shRNA specifically targeting the *ATG9A* mRNA (target sequence localized in the exon 16). We then selected and analyzed several puromycin-resistant clones (sh-*ATG9A*; clones A, B, and C). These clones exhibited a strong decrease in *ATG9A* mRNA levels (Figure 3A) as well as protein levels (Figure 3B,C), when compared to the control cell lines transfected with a vector coding a scramble shRNA (sh-*control*; clones 1 and 2). We then investigated whether a decrease in *ATG9A* expression in the MDA-MB-436 cell line would alter its tumor-linked features such as in vitro proliferation and invasion. We first analyzed cell proliferation by performing a MTT assay and a cell viability assay (trypan-blue exclusion test). The MTT results indeed showed that the sh-*ATG9A* cell lines exhibited a strong decrease in proliferation compared to the control cell lines. The trypan-blue exclusion assay confirmed these data since we observed a significant decrease in cell number, but no significant increase in cell death (Figure 3D,E). In order to confirm the pro-tumoral properties of ATG9A, we quantified the invasion ability of our clones using Boyden chambers. We indeed observed that the invalidation of *ATG9A* expression strongly decreased cell invasion (Figure 3F). Altogether, these data confirmed that a decrease in ATG9A expression led to the inhibition of pro-cancer phenotypes, and were therefore coherent with our in vivo data showing a high expression of ATG9A in TNBC.

We then decided to inhibit *ATG9A* expression in a second TNBC cell line, MDA-MB-231. Our results showed that we indeed inhibited *ATG9A* mRNA levels as well as protein levels, but we did not observe any change in cell proliferation or migration in the sh-*ATG9A* cells compared to the control cells (Figure S3). This could be explained by the different origin and genomes of these two cell lines or their mutation burden as we also established that this MDA-MB-231 cell line seemed to be less sensitive to changes in autophagy flux (Figure S4). Indeed, this cell line presented lower basal LC3B-II levels than the MDA-MB-436 ones, and these levels remained lower than the ones observed in the MDA-MB-436 cell line after Baf A1 incubation, suggesting a lower autophagy flux in the MDA-MB-231 cell line (Figure S4A,B). Moreover, this cell line was less sensitive to inhibition of autophagy since their proliferation was not altered after incubation with 3-MA, an inhibitor of the early steps of autophagy (Figure S4C,D). We also observed that the proliferation rate of MDA-MB-231 cells was not modified after a knock-down of *ATG9A* together with an incubation of 3-MA, while MDA-MB-436 cell proliferation was decreased in the sh-*ATG9A* cells compared to the controls, but did not further decrease after the addition of 3-MA, suggesting that the autophagy flux had already highly decreased in these cells (Figure S4E,F).

**Figure 4:**
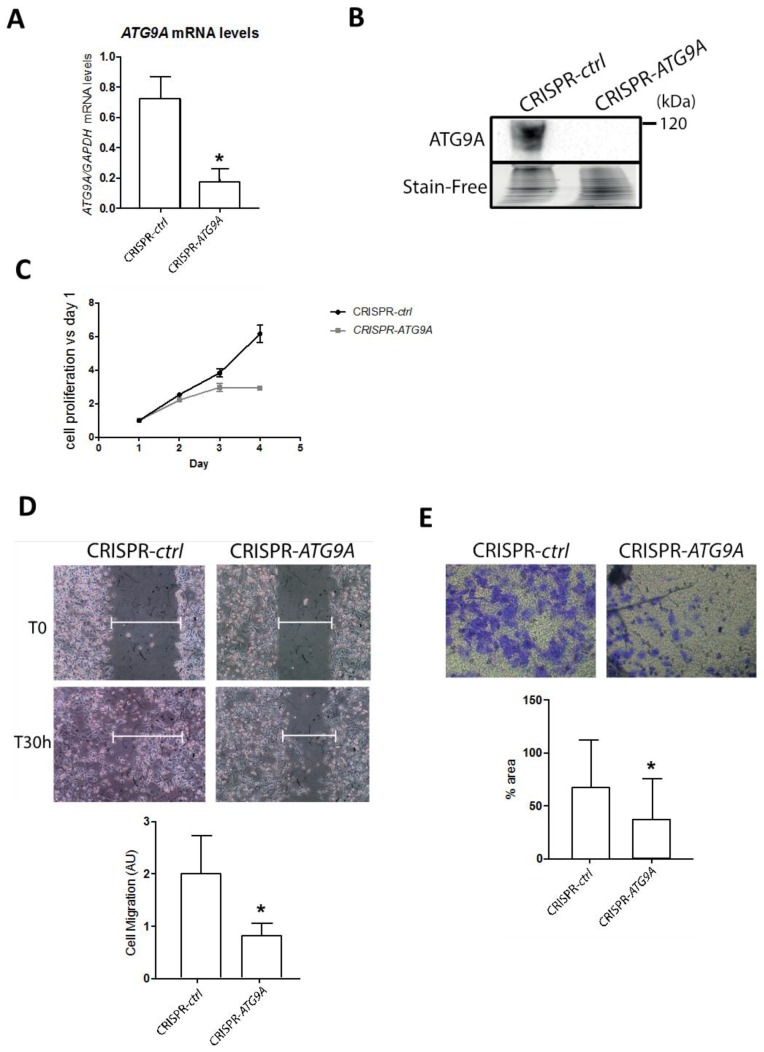
Inhibition of ATG9A expression using CRISPR-Cas9 inhibits in vitro cell proliferation, migration, and invasion in the TNBC cell line MDA-MB-436. (**A**) ATG9A mRNA expression levels quantified by RT-qPCR in the CRISPR-*control* or the CRISPR-*ATG9A* clones (*n* = 3). Values were normalized using the housekeeping gene *GAPDH*. The difference of expression was quantified using a t-test. * *p* ≤ 0.05. (**B**) Level of expression of the ATG9A protein in the CRISPR-*control* and the CRISPR-*ATG9A* clones were analyzed by western blotting. A representative image of three independent experiments is shown. (**C**) The proliferation rates of the CRISPR-*ATG9A* clone and its control were quantified using a MTT assay conducted every day over a 4 day period (*n* = 3, 16 replicates). The difference in cell proliferation was quantified using a t-test at day 4. *p* ≤ 0.05. (**D**) The migration ability of the CRISPR-*ATG9A* and -*control* clones were analyzed thanks to a wound healing assay. A representative image of three independent experiments is shown. The difference in cell migration was quantified using a t-test. * *p* ≤ 0.05. (**E**) The invasion capacity of the CRISPR-*ATG9A* clone and -*control* clone was performed in Boyden chambers. A representative image of three independent experiments is shown. The graph represents the percentage of cells which migrated through the ECM-coated Boyden chambers after 24 h, quantification done with the ImageJ software. The difference in cell density was quantified using a t-test. * *p* ≤ 0.05.

Since a very low residual protein expression was still detected in our MDA-MB-436 shRNA-designed cell lines, and that shRNA had been described to sometimes produce off-target effects, even if we compared three independent clones. Next, we decided to confirm our results in a genetic model constructed using the CRISPR-Cas9 technology. We therefore transfected MDA-MB-436 cells with the pSpCas9(BB)-2A-GFP vector, which encodes the Cas9-GFP protein as well as a sgRNA (single guide RNA) designed to specifically target the *ATG9A* gene (target sequence localized in the exon 8). Following transfection, the GFP-positive cells were selected and analyzed for *ATG9A* expression. *ATG9A* mRNA was significantly decreased in the CRISPR-*ATG9A* clone compared to the control cells (Figure 4A). Indeed, mRNA with premature non-sense mutations or premature termination codon (PTC), which are frequently observed in genetic diseases or with the CRISPR technology, are generally eliminated by the non-sense mediated mRNA decay pathway (NMD) [24]. We next confirmed the absence of the ATG9A protein in the CRISPR-*ATG9A* cells (Figure 4B). To determine whether the genetic invalidation of ATG9A expression also led to an inhibition of cancer-associated phenotypes, similar to the ones already observed in the sh-*ATG9A* clones, we quantified the proliferation rate of these cells using a MTT assay (Figure 4C). As expected, CRISPR-*ATG9A* cells presented a lower proliferation rate than control cells. Moreover, these cells also showed significant invasion capacities compared to control cells (Figure 4D,E).

## 4. Discussion

Triple negative breast cancer is an aggressive subtype with limited treatment options and very poor prognosis. Finding new biomarkers may be useful for better and earlier diagnosis, but might also lead to the characterization of novel targets for therapy. Since many studies have shown a link between autophagy and breast cancer (reviewed in [25]), this pathway may represent a new way of treatment, but the role of autophagy in cancer still remains largely unclear. On one hand, it is widely accepted that an inhibition of autophagy may increase the apparition of genetic mutations and therefore favor the apparition of cancer cells. On the other hand, it has also been described that autophagy can enhance cancer cell survival, since autophagy can support cancer cell survival in response to various stresses, such as starvation or hypoxia, or even induce anti-cancer drug resistance [4,26]. Therefore, further studies will be needed to clear out this paradox and determine the potential of autophagy as an anti-cancer target. Indeed, the role of ATG9A in autophagy seems dependent on the in vitro model or the type of cancer analyzed, and appears complex in cancers. For example, invalidation of *ATG9A* in MEFs (Mouse Embryonic Fibroblasts) provoked a significant decrease in LC3B-II accumulation in profit of LC3-I [10]. In contrast, knockdown of *ATG9A* in pancreatic cancer cells induced an accumulation of the LC3B-II form [27]. Similar observations to the latter case were observed in our invalidation model of *ATG9A* in MDA-MB-436 cells, since we observed LC3B-II accumulation and therefore autophagy induction or inhibition of autophagosome degradation (Figure S5). Nevertheless, clinical trials are already underway to test the effect of inhibitors (hydroxychloroquine) or inducers of autophagy (Everolimus), alone or in combination with various chemotherapies (see the *Clinicaltrials.gov* website for more information; reviewed in [28]).

The first question to ask when willing to target the autophagy pathway, is whether autophagy is increased or inhibited in tumors compared to controls, but to get the right assessment, researchers are still looking for reliable autophagy biomarkers or new targets. For example, an increase in LC3B protein staining within tumor cells, which can be directly associated to an elevated number of overall autophagosomes within the cells, can be the consequence of increased autophagy flux or decreased degradation of autophagosomes by lysosomes. This is why our study aims at analyze the expression levels of different autophagy genes in a cohort of breast cancers. Our data showed, for the first time, that one autophagy gene, *ATG9A*, presented a significant increase in triple negative breast cancer patients compared to healthy adjacent tissues and correlated with the expression of the proliferative marker KI67. Moreover, using available bioinformatics databases, such as the survival database “KM plotter”, we observed that high *ATG9A* levels correlated with a significantly shorter relapse-free survival (RFS). We then developed in vitro *ATG9A* invalidation models in the TNBC cell line MDA-MB-436 and showed that a decrease in ATG9A expression led to an inhibition of cancer phenotypes within these cells. These in vitro data confirmed that ATG9A was necessary for the aggressive character of this cell line and that a high expression of this gene might be considered as a biomarker of TNBC even if ATG9A was also expressed at a lower level in LumA cancers. Nevertheless, it would be interesting to analyze a greater number of TNBC cases and follow *ATG9A* expression before, during, and after treatment to determine whether this gene could also become a marker of treatment efficiency or outcome.

These data are partly contradictory with the ones published recently, which showed that *ATG9A* mRNA expression was significantly decreased in invasive ductal carcinomas compared to matched healthy tissues [18]. However, when the authors analyzed *ATG9A* mRNA levels and correlated them with clinicopathological characteristics (ER, PR, or HER2 status), they only demonstrated a significantly lower *ATG9A* expression in the HER2^+^ subtype of breast cancers. Our results were therefore consistent with these conclusions since we also observed, in our cohort, a decrease in *ATG9A* mRNA expression in HER2^+^ patients, even if this decrease was not significant, mainly due to the low number of samples of this subgroup in our cohort (*n* = 6). Moreover, it is noteworthy that autophagy genes or protein levels can vary according to the cancer grade or even the cancer subtype analyzed. For example, we previously reported that the levels of *GABARAPL1*, a member of the ATG8 family necessary for autophagosome elongation and closure, decreased in numerous cancer cell lines and that a high expression of this gene, in a cohort of 256 samples, correlated with good prognosis in patients with breast cancers [29,30]. Nevertheless, another laboratory showed that *GABARAPL1* was up-regulated in TNBC cell lines and tissue, and that a high expression in this gene was associated with shorter overall survival [31], suggesting that the analysis of mRNA expression using BC molecular subtypes could lead to significantly different conclusions, compared to a non-classified report, thus proving the heterogeneity of BC. Here, we show that similarly to *GABARAPL1* [30], *ATG9A* was up-regulated in TNBC and its inhibition decreased cancer phenotypes and abilities. Altogether, these data suggest that the expression of some ATGs may vary according to the type of cancer and the subtype analyzed, and that the decision to target the autophagy pathway as a therapy might be restricted to some type of cancer or even to a small number of patients, confirming the importance of establishing strong and reliable autophagy biomarkers.

Regarding ATG9A, the conclusions on its expression and function in cancer can also vary according to the study. For example, it has been recently shown that TMEM74 (Transmembrane 74) is able to induce autophagy and favor cancer cell survival via its interaction with ATG9A and ATG16L1 [32], and that a high expression of TMEM74 is linked to a decrease of patient survival in different cancers, suggesting that ATG9A together with TMEM74 would be a factor of poor prognosis. Another study showed that *miR-29a*, which targets the *ATG9A* and *TFEB* mRNAs, is down-regulated in Pancreatic Ductal Adenocarcinoma (PDAC) leading to increased *ATG9A* levels, autophagy flux and resistance to gemcitabine, as well as increased cancer cell migration. A third study described that ATG9A was induced in response to hypoxia in glioblastoma (GBM) and that ATG9A depletion led to decreased in vitro proliferation as well as delayed in vivo tumor growth [33]. These data are therefore in agreement with ours, describing a pro-tumor role of *ATG9A* in cancers. In contrast, it has been recently suggested that the long non-coding RNA (lncRNA) *HAGLROS* was induced in gastric cancers and that its down-regulation led to decreased migration and invasion of gastric cancer cells, but also to increased *ATG9A/B* levels [34], suggesting an anti-tumor role of ATG9A.

Taken together, our results highlight, for the first time, the clinical relevance of *ATG9A* as a therapeutic target in triple negative breast cancer patients. However, future work will be necessary to characterize the molecular mechanisms leading to cancer-related phenotypes associated to *ATG9A*. Amongst the cancer-related phenotypes, stemness is determined by the capacities of cells to proliferate in serum free conditions. Indeed, cancer stem cells (CSCs) are supposed to play a key role in cancer relapse due to their high resistance to treatments and their high survival rate in low nutriment microenviroment. Tumorsphere assays have already been used in order to determine the role of autophagy in CSC proliferation or survival, but as seen above for other cancer-related phenotypes, contradictory effects have been reported depending of the cancer studied. For example, a combination of radiotherapy with chloroquine in U87 cells (glioma-derived cells), to inhibit radiation-induced autophagy, significantly decreased tumorsphere formation, suggesting a positive role of autophagy on tumorsphere survival [35]. In contrast, the JAK2 (Janus Kinase) inhibitor SAR317461, which induces autophagy, led to a decrease of tumorsphere numbers suggesting an inhibitory role in autophagy [36]. Therefore, it is clear that further studies would be needed to determine whether ATG9A could modify the ratio of stem cells vs. cancer cells in TNBC.

We have previously demonstrated that another autophagy gene, *GABARAPL1*, presents anti-tumor effects but we also recently described that the expression of a G116A mutant of GABARAPL1, which cannot be conjugated onto autophagosomes and therefore to participate in autophagosome elongation and maturation, was still able to decrease tumor growth in vivo [20], suggesting that the anti-tumor properties of *GABARAPL1* may not only be linked to its role in autophagy. Therefore, it would be of great interest to determine whether the role of ATG9A in TNBC is also linked to its role in autophagy.

## Figures and Tables

**Table 1:** Clinical information regarding the patients included in the study.

Cohort 1 (37)		Cohort 2 (80)	
Characteristics	N	Characteristics	N
Age (year) (37)		Age (year) (80)	
>57	18	>58	40
≤57	19	≤58	40
BC subtype (37)		BC subtype (80)	
LumA	13	LumA	30
LumB	10	LumB	14
HER-2	4	HER-2	6
TN	10	TN	30
Grade (36)		Grade (79)	
I	5	I	9
II	21	II	37
III	10	III	33
pT (mm) (35)		pT (mm) (78)	
≤20	18	≤20	35
20–50	16	20–50	41
≥50	1	≥50	2
pN (36)		pN (80)	
negative	17	negative	41
positive	19	positive	39
ER status (37)		ER status (80)	
negative	14	negative	36
positive	23	positive	44
PR status (37)		PR status (80)	
negative	15	negative	39
positive	21	positive	41
HER-2 status (37)		HER-2 status (80)	
negative	23	negative	60
positive	14	positive	20

The table on the left represents Cohort 1, which was used to quantify the expression of *ATG9A*, *ATG9B*, *BECLIN1*, *LC3B*, *NIX*, and *P62/SQSTM1* mRNA levels. The table on the right (Cohort 2) represents the extended cohort, which was used to further analyze *ATG9A* mRNA levels in BC. N: Number of patients; the numbers in brackets are the total number of analyzed samples among the cohort; pT: Tumor size; pN: Lymph node status; ER: Estrogen receptor; PR: Progesterone receptor; HER-2: Human epidermal growth factor receptor-2.

**Table 2:** Primer sequences used for Syber Green RT-qPCR experiments.

Gene	Forward	Reverse
18S rRNA	5’-GTAACCCGTTGAACCCCATT-3’	5’-CCATCCAATCGGTAGTAGCG-3’
ATG9A	5’-TTTGCGTTAGGGTGAAGACC-3’	5’-AGGGCAGCAAAGTATTTCCA-3’
ATG9B	5’-CCTTGGGCAGTTCTTCTTTG-3’	5’-CTTCCTGGTGCCTGGTACAT-3’
BECLIN1	5’-TCACCATCCAGGAACTCACA-3’	5’-CCTGGCGAGGAGTTTCAATA-3’
LC3B	5’-CGGAAAGCAGCAGTGTACCA-3’	5’-GGCAGAAGGGAGTGTGTCTGA-3’
NIX	5’-AAGGCAGGCTTCATTTTTCA-3’	5’-CCAATAATTTCCACAACGGG-3’
RPLP0	5’-TCGACAATGGCAGCATCTAC-3’	5’-GCCTTGACCTTTTCAGCAAG-3’
SQSTM1	5’-ATCGGAGGATCCGAGTGT-3’	5’-TGGCTGTGAGCTGCTCTT-3’

## Supplementary Materials

The following are available online at http://www.mdpi.com/2073-4409/7/12/248/s1, Figure S1: ATG9A protein expression quantified in five different TNBC cell lines; Figure S2: ATG9A protein expression using IHC; Figure S3: *ATG9A* extinction in the MDA-MB-231 cell line does not lead to changes in cancer-linked phenotypes; Figure S4: The MDA-MB-231 cell line is less sensitive to autophagy inhibition than MDA-MB-436 cells; Figure S5: *ATG9A* extinction led to an increase in LC3B-II levels in the MDA-MB-436 cell line.

## Abbreviations

ATG	autophagy-related
BC	breast cancer
CSCs	cancer stem cells
DMEM	dulbecco’s minimum essential medium
EBSS	earle’s balanced salt solution
ECM	extra cellular matrix
ER	estrogen receptor
FBS	fetal bovine serum
HER2	Human Epidermal Growth Factor Receptor 2
HRP	horseradish peroxidase
IHC	immunohistochemistry
LC3B	microtubule-associated protein 1 light chain 3B
mTORC1	mammalian target of rapamycin complex 1
MTT	3-(4,5-dimethylthiazol-2-yl)-2,5-diphenyltetrazolium bromide
PBS	phosphate buffer saline
PBS-T	PBS triton-X-100
pN	lymph node status
PR	Progesterone Receptor
pT	tumor size
PVDF	polyvinylidene difluoride
RT-qPCR	reverse transcription-quantitative polymerase chain reaction
SDS	sodium dodecylsulfate
sgRNA	single guide RNA
SISH	silver in situ hybridization
TN	triple negative
TNBC	triple negative breast cancer
