# Supplementary material for: ATG9A Is Overexpressed in Triple Negative Breast Cancer and Its In Vitro Extinction Leads to the Inhibition of Pro-Cancer Phenotypes
Source: Cells. 2018 Dec 6;7(12):248. doi: 10.3390/cells7120248 (PMC6316331; doi:10.3390/cells7120248)
Supplement: Supplementary file 1 [file cells-07-00248-s001.pdf]

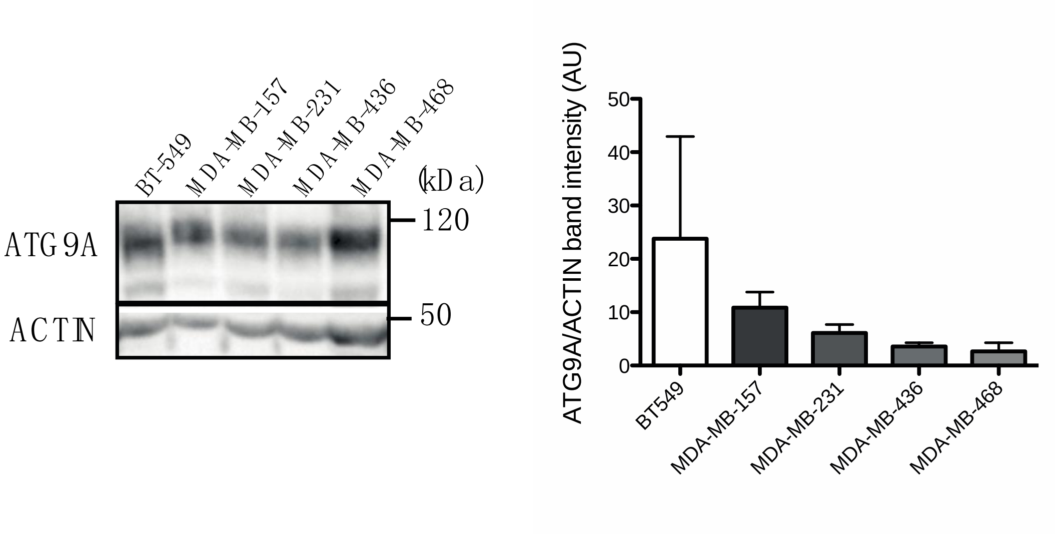

Supp. Figure S1. ATG9A protein expression quantified in five different TNBC cell lines. A representative image of 10 independent experiments is shown on the left. The histograms on the right represent the ATG9A protein signals quantified with the ImageLab software and normalized to ACTIN protein levels, (n = 10).

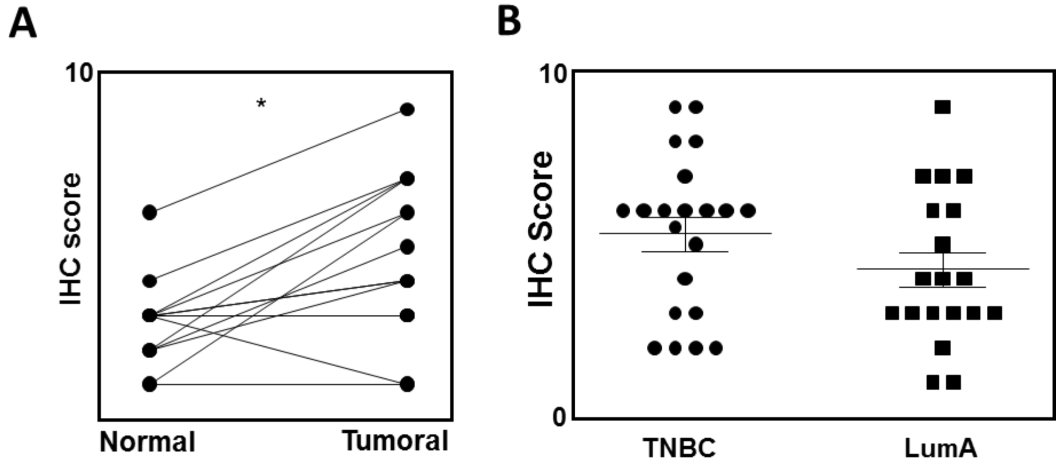

Supp. Figure S2. ATG9A protein expression using IHC. (A) Quantification of ATG9A protein levels using IHC in LumA tumors compared to normal adjacent tissue. (B) Comparison of expression of ATG9A between triple negative breast cancer (TNBC) and Luminal A biopsies.

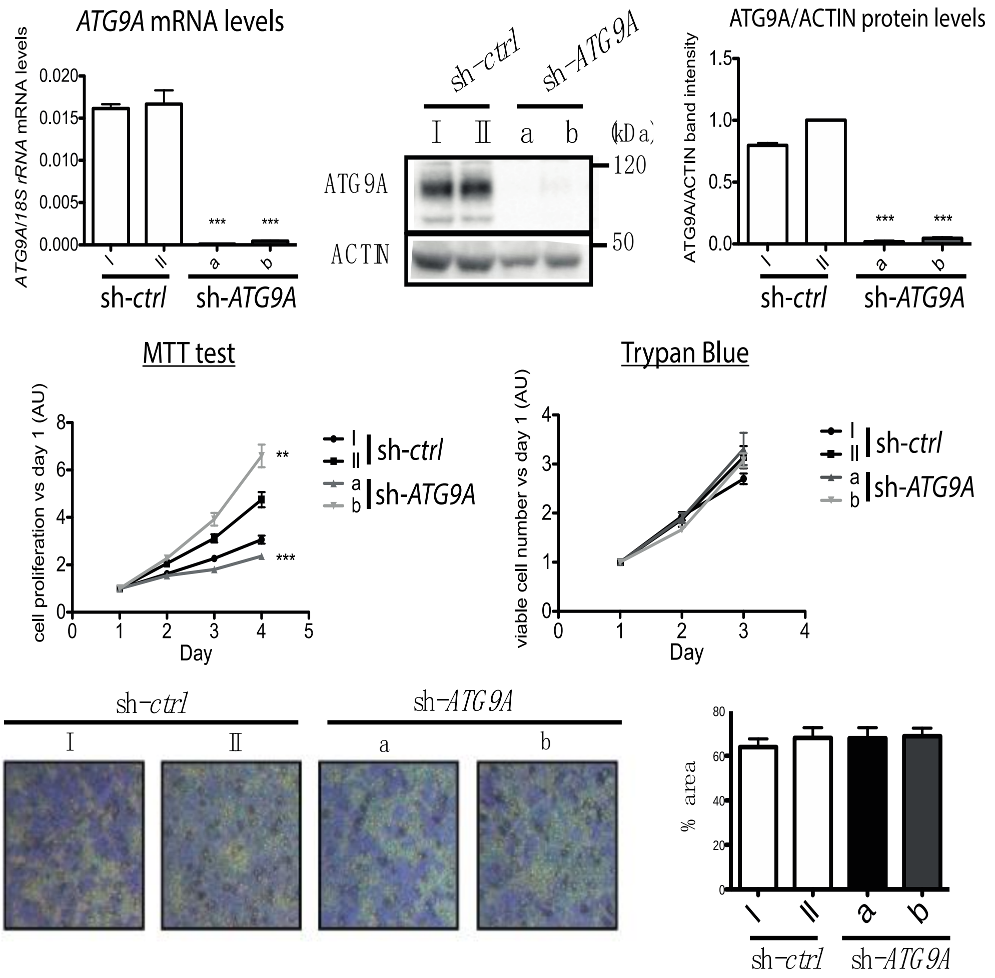

Supp. Figure S3. *ATG9A* extinction in the MDA-MB-231 cell line does not lead to changes in cancer-linked phenotypes. **A)** ATG9A mRNA expression levels quantified by RT-qPCR in stable MDA-MB-231 clones expressing a sh-*ctrl* (I and II) or a sh-*ATG9A* (a and b) (n = 3, duplicate). Values were normalized using the housekeeping gene *rRNA18S*. Difference of expression was quantified using a t-test: compared to sh-*ctrl* I or II, $p<0.0001$ for all sh-*ATG9A* clones (a, b); **B)** Levels of ATG9A and ACTIN proteins in the sh-*ctrl* (clones I and II) or sh-*ATG9A* (clones a and b) clones. A representative image of 3 independent experiments is shown. **C**) Quantification of ATG9A protein levels normalized with ACTIN protein levels, quantified using the ImageLab software (n = 3). The difference of expression was determined using a t-test: compared to sh-*ctrl* I or II, $p < 0.0009$ for sh-*ATG9A* clones a and b. **D**) Proliferation rates of sh-*ATG9A* and their controls using a MTT or **E**) a Trypan-blue exclusion assay. The MTT assay was conducted every day over a 4-day period (n = 3, 16 replicates). The difference in cell proliferation was quantified using a t-test at day 4, compare to sh-*ctrl* I. ***: $p \leqslant 0.001$. **: $p \leqslant 0.01$. For the trypan blue exclusion assay, viable cells were counted every day over a 3-day period (n = 3, 4 replicates). No significant difference in cell number versus day 1 was quantified using a t-test at day 3 (graphs on the right), compare to sh-*ctrl* I or II. **F**) The invasion assay was performed using sh-*ATG9A* (a and b) and control clones (I and II). A representative image of 3 independent experiments with duplicates is shown on the left. The graph on the right represents the percentage of cells that migrated through the ECM-coated Boyden chambers after 24 h, quantification done with the ImageJ software.No significant difference has been quantified using a t-test, compate to sh-*ctrl* I or II.

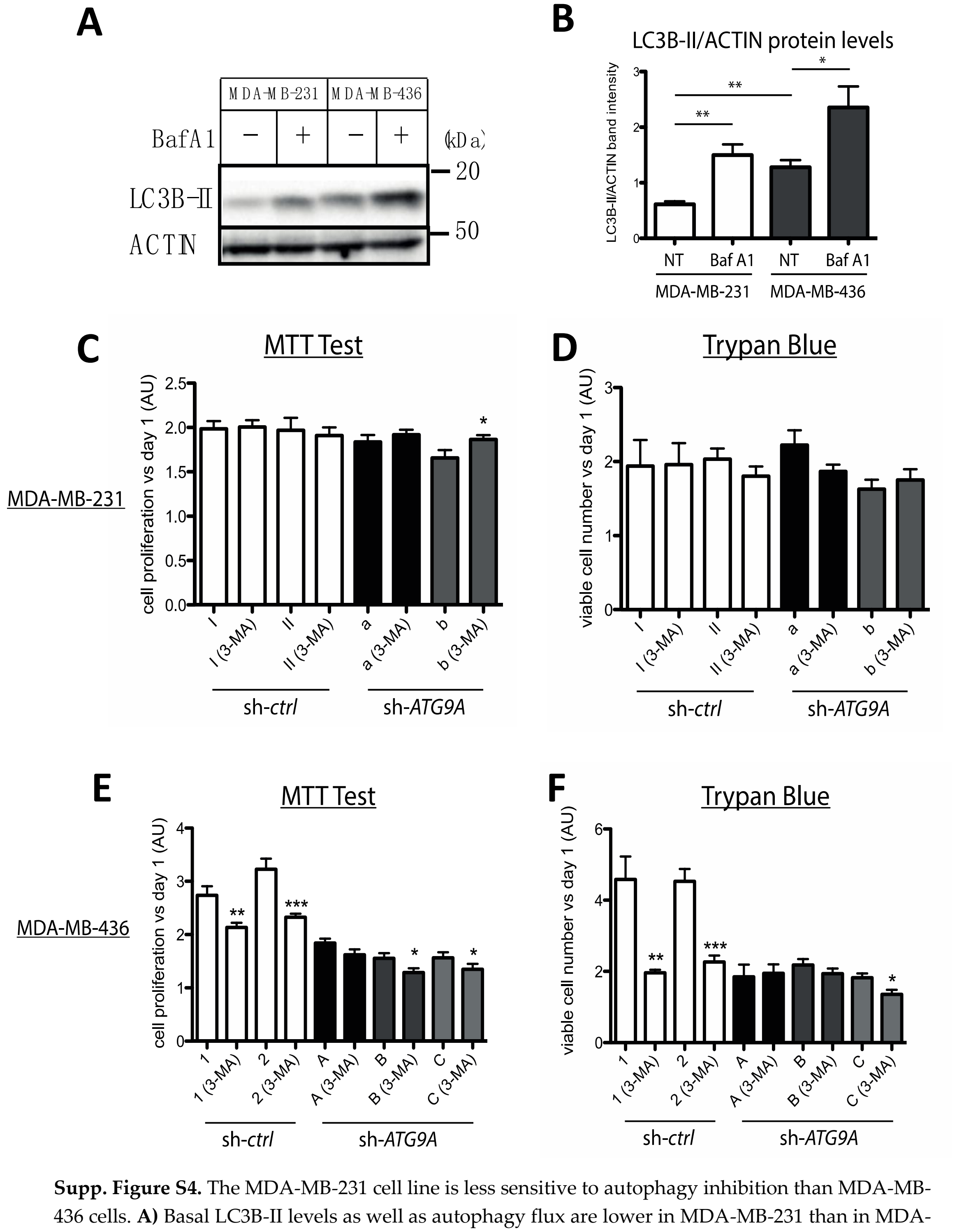

Supp. Figure S4. The MDA-MB-231 cell line is less sensitive to autophagy inhibition than MDA-MB-436 cells. **A)** Basal LC3B-II levels as well as autophagy flux are lower in MDA-MB-231 than in MDA-MB-436. Levels of LC3B-II and ACTIN proteins in MDA-MB-231 than in MDA-MB-436 cell lines were quantified in the presence or absence of Bafilomycin A1 (Baf A1). A representative image of 3 independent experiments is shown on the left. **B)** The histograms represent the LC3B-II protein signals quantified with the ImageLab software and normalized to ACTIN protein levels, (n = 3). **C)** and **D)** Incubation of MDA-MD-231 (sh-*ctrl* and -*ATG9A*) and **E)** and **F)** MDA-MB-436 cell lines (sh-*ctrl* and -*ATG9A*) with 3-MA (72 h, 2.5 mM) led to an inhibition of MDA-MB-436 cell proliferation observed by **E)** MTT test at day 4 compare to day 1 and **F)** Trypan Blue assay at day 3 compare to day 1. MDA-MB-231 cells did not present any change in proliferation rate using a **C)** MTT test at day 4 and **D)** Trypan Blue assay at day 3 compare to day 1. Moreover, addition of 3-MA in MDA-MB-231 sh-*ATG9A* clones still did not down-regulate cell proliferation and did not further reduce MDA-MB-

231 sh-*ATG9A* clone cell proliferation. The difference in cell proliferation after 3-MA treatment was quantified using a t-test, compare to non-treated cells. ***: p ⩽ 0.001. **: p ⩽ 0.01. *: p<0.05.

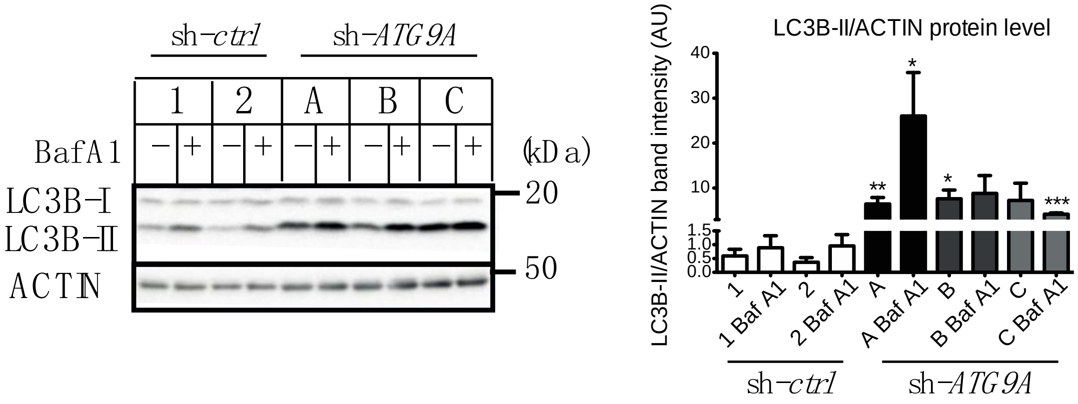

Supp. Figure S5. *ATG9A* extinction led to an increase in LC3B-II levels in the MDA-MB-436 cell line. Levels of LC3B and ACTIN proteins in sh-*ctrl* (1 and 2) and sh-*ATG9A* (A, B and C) clones were quantified in the presence or absence of Bafilomycin A1 (Baf A1). A representative image of 4 independent experiments is shown on the left. The histograms on the right represent the LC3B-II protein signals quantified with the ImageLab software and normalized to ACTIN protein levels (n = 4). The difference in normalized LC3B-II levels were quantified using a t-test. ***: p ⩽ 0.001. **: p ⩽ 0.01. *: p<0.05.
